# First morphological description of the Galápagos pink iguana (*Conolophus marthae*) hatchling: a critical step for its conservation

**DOI:** 10.7717/peerj.20683

**Published:** 2026-01-27

**Authors:** Jorge Carrión-Tacuri, Christian Sevilla, Jean Pierre Cadena-Murillo, Willians Castro, Walter Chimborazo, Adrián Cueva, Cristian Gil-Jaramillo, Roberto Jiménez Carrión, Janaí Yépez Ruiz, Gregory A. Lewbart, Diego Páez-Rosas, James P. Gibbs

**Affiliations:** 1 Fundación Conservando Galápagos, Puerto Ayora, Galápagos, Ecuador; 2 Galápagos Conservancy, Washington, District of Columbia, United States; 3 Dirección del Parque Nacional Galápagos, Puerto Ayora, Galápagos, Ecuador; 4 North Carolina State University, Raleigh, North Carolina, United States; 5 Universidad San Francisco de Quito, Puerto Baquerizo Moreno, Galápagos, Ecuador; 6 State University of New York (SUNY), Syracuse, New York, United States

**Keywords:** Pink iguana, *Conolophus marthae*, Hatchling, Ontology, Morphology, Camouflage, Galapagos Islands

## Abstract

The Galapagos pink iguana (*Conolophus marthae*) is endemic to Wolf Volcano on Isabela Island in the Galapagos archipelago. Due to its remote and hard-to-access habitat, the recently discovered and critically small wild population of the pink iguana has been extremely challenging to study. Herein we provide a first description of the morphology and behavior of six *C. marthae* hatchlings, and compare them with 12 hatchlings of the sympatric *C. subcristatus*. Morphometric measurements (snout–vent length = 10.9 ± 1.63 (SD) cm, tail length = 17.9 ± 3.05 cm, and weight = 47.8 ± 25.4 g) revealed a longer tail relative to its body size (ratio = 1.65 ± 0.23) compared to the sympatric Galápagos land iguana (*C. subcristatus*) hatchling of similar size (ratio = 1.42 ± 0.11). *C. marthae* hatchlings also displayed distinctive coloration with a bright green dorsal background with irregular black maculations and a pale, nearly unpigmented ventral surface. A comparative photograph of a subadult *C. marthae* revealed a directional, ontogenetic color shift: green dorsal areas became black while black maculations gave rise to pink patches, possibly a retained ancestral trait with implications for camouflage or signaling. These findings fill a knowledge gap in the early ecology of Galápagos pink iguana, providing information useful for monitoring recruitment in this Critically Endangered species.

## Introduction

The Galápagos pink land iguana (*Conolophus marthae*) is one of three species of terrestrial iguanas endemic to the archipelago (Gentile & Snell, 2009). Although formally described in the scientific literature only recently (Gentile & Snell, 2009), *C. marthae* represents the oldest extant lineage within the genus *Conolophus* (Paradiso et al., 2025). Mitochondrial and nuclear DNA analyses have revealed that *C. marthae* is deeply divergent from all other *Conolophus* species (Gentile et al., 2009) and, as such, the most basal lineage within the Galápagos land iguanas (Tzika et al., 2008, López-Delgado et al., 2025). This basal position provides an exceptional opportunity to investigate ancestral morphological traits and color patterns within the genus, as ontogenetic processes in related iguanid taxa have been linked to both ecological adaptation and phylogenetic conservation (Rosenblum, 2005; Bagnara, Fernandez & Fujii, 2007; Gentile & Snell, 2009).

*C. marthae* species is currently listed as Critically Endangered (Gentile, 2012), and restricted to only a single population estimated at 189 adults (95% CI [150–270]; Garizio et al., 2024) confined to a small area on the northwestern slope of Wolf Volcano of Isabela Island (Gentile & Snell, 2009; Gentile et al., 2016; Gargano et al., 2024). Despite its conservation status and evolutionary significance, many aspects of the life history, particularly reproduction, of *C. marthae* remain poorly understood. In particular, little is known about the reproductive biology and early developmental stages of *C. marthae*, and no information existed on hatchling morphology or behavior prior to this study. The pink iguana’s nesting area was first located in 2021 on the northern flank of Wolf Volcano using strategically deployed camera traps (Galápagos Conservancy, unpublished data), which enabled documentation of nesting activity. However, the hatchlings described in this study were not captured at nests; instead, most were found opportunistically in the summit area during independent field surveys. The first hatchlings of *C. marthae* were located and captured during iguana monitoring activities in 2022 (Cadena-Murillo et al., 2023; Ramírez-Kastdalen, Cadena-Murillo & Ortiz-Catedral, 2023).

The main objective of this study is to provide the first detailed characterization of the external morphology, coloration, and behavioral traits of *Conolophus marthae* hatchlings, establishing a baseline for future population monitoring. Until now, no hatchling had ever been described for any Galápagos land iguana species, leaving a critical gap in understanding early life stages and recruitment dynamics. Here we provide the first description of *C. marthae* hatchlings, characterizing their external morphology, coloration, and behavioral responses during handling. We also visually examine color transition from hatchling to subadult life stage to provide insight into ontogenetic changes and adaptive significance. Understanding hatchling morphology and behavior is essential for interpreting early-stage survival and recruitment, processes that ultimately determine the long-term viability of this critically endangered species. These observations can help guide conservation actions prioritized in the management plan for this species, whose primary threat is depredation by introduced mammals (*i.e*., feral cats and rodents) on hatchlings and juveniles and for which recruitment monitoring is essential.

## Materials and Methods

### Ethics statement and animal care

This study was supported and conducted by the Galápagos Conservancy (GC) and the Galápagos National Park Directorate (GNPD) under research permit PC-77-24. The fieldwork and data collection were carried out following the protocols of ethics and animal handling NC State IACUC #23/157. All animals involved in this study were wild and not under human care and monitored by a veterinarian and a GNPD rangers during this study.

### Fieldwork and animal selection

**Figure 1:**
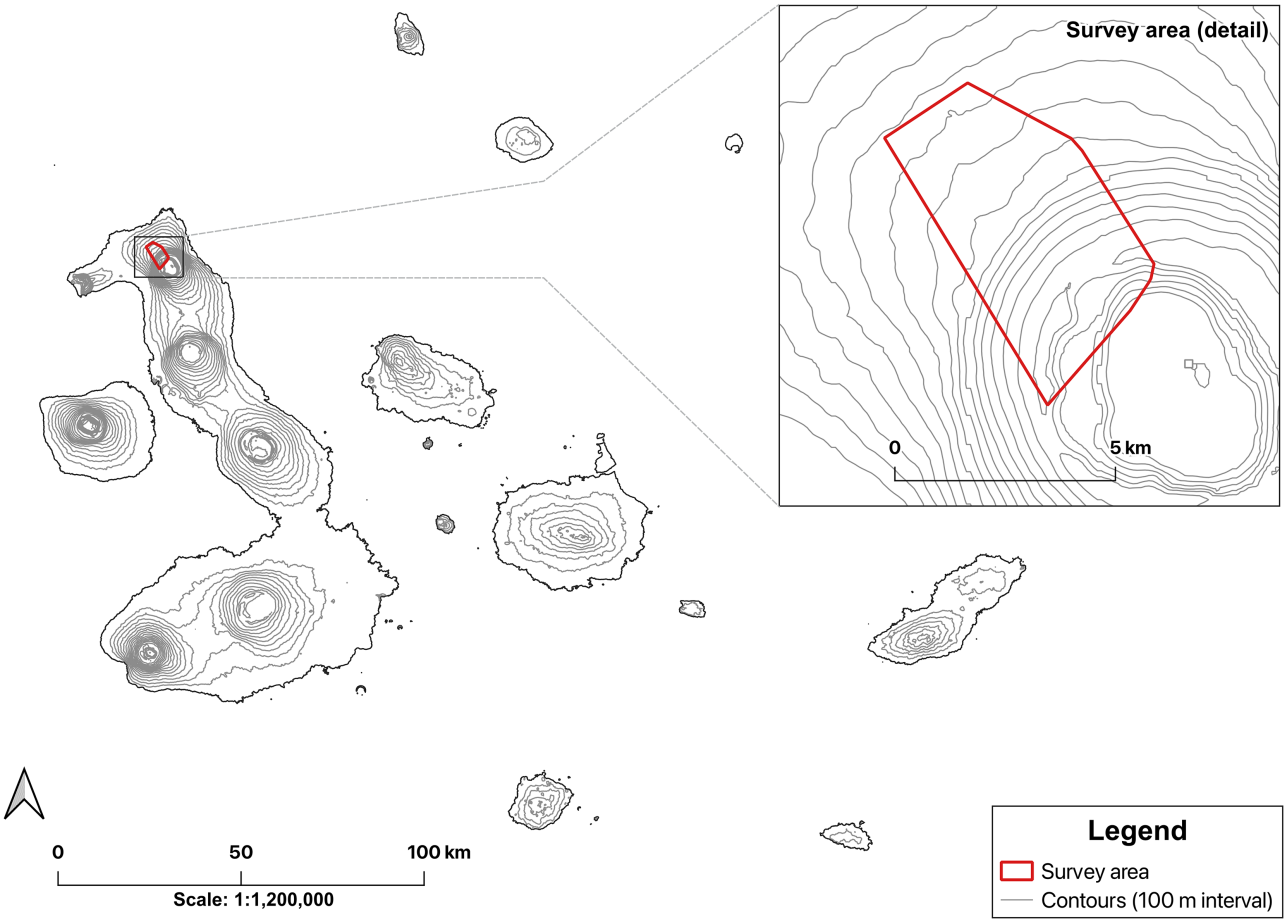
Distribution of surveyed area on Wolf Volcano. Location of Wolf Volcano, Isabela Island, Galápagos, and survey area (marked) where hatchlings of *Conolophus marthae* and *C. subcristatus* were encountered during 2022 and 2025 field expeditions.

Observations of *C. marthae* were made during field expeditions in 2022 and 2025. Searches were carried out during daylight hours across the crater rim, inner caldera slopes, and upper flanks of the volcano (Fig. 1), covering the known activity area of *C. marthae*. We concentrated our search efforts near the only known nesting zone and places where the first hatchlings were captured (Cadena-Murillo et al., 2023; Ramírez-Kastdalen, Cadena-Murillo & Ortiz-Catedral, 2023). Individuals were located by visual encounter and opportunistic detection during daily ground surveys. Six hatchlings of *C. marthae* and twelve of *C. subcristatus* were captured by hand and processed *in situ*.

### Morphometric data collection

At time of capture, morphological measurements included snout–vent length (SVL), tail length (TL), and mass were collected in the field with flexible tape and spring scale Micro-Line 20100, 1,000 g capacity in 1 g increments. Hatchling coloration of both *C. marthae* and *C. subcristatus*, the only two land iguanas present at the site, were documented visually and with high-resolution photographs (4,032 × 3,024 px, 12 MP) from multiple perspectives (*i.e*., dorsal, lateral and ventral perspectives). For ontogenetic comparison, visual and photographic (natural light, without flash or enhancement) records were made for *C. marthae* hatchlings and subadults, focusing on relative positions and transitions of pigmented areas, with attention to dorsal and lateral zones. Finally, behavioral responses of hatchlings for both species were assessed qualitatively based on their immediate reaction to human approach. Observations include the perceived quickness of escape attempts, the tendency to seek refuge, and the relative ease or difficulty to escape under equivalent search effort. Encounters occurred on the summit of Wolf Volcano, a dry, sparsely vegetated habitat under clear to cloudy conditions. Considering individuals were detected at varying distances and states of alertness, it was not possible to determine latency to flee or quantify escape attempts consistently. Moreover, handling time of hatchlings captured was intentionally kept to a minimum to avoid inducing stress, making recording of comprehensive morphological measurements infeasible.

## Results

### Morphological comparison

**Figure 2:**
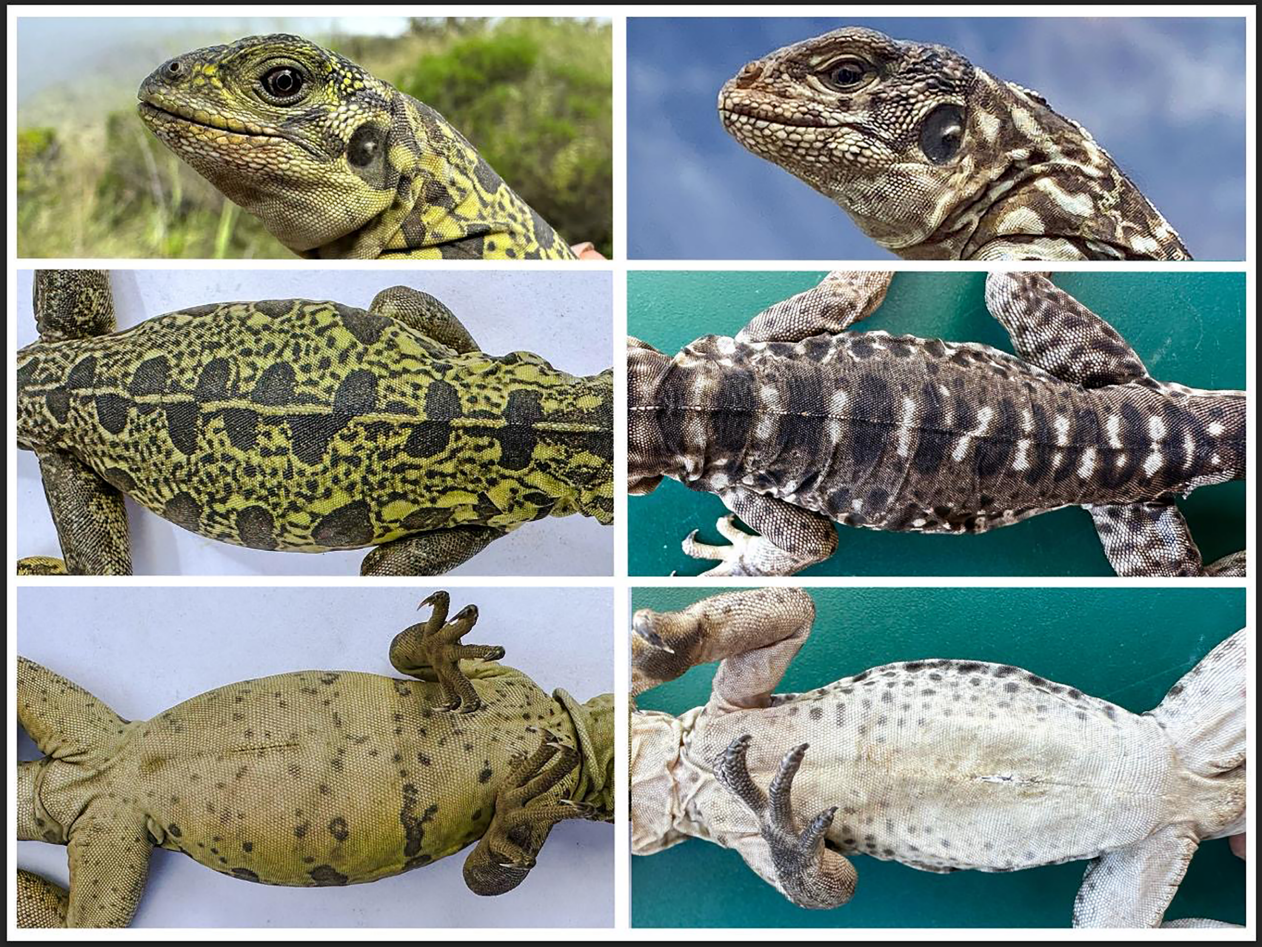
Dorsal and ventral morphology of hatchlings. Dorsal and ventral views of hatchlings of *Conolophus marthae* (left panels) and *C. subcristatus* (right panels). Photographs show contrasting color patterns between species and differences in ventral pigmentation. Pictures taken under natural light without enhancement. (Photo credit: Jorge Carrión-Tacuri).

The *C. marthae* hatchlings (*n* = 6) measured 10.9 ± 1.63 (SD) cm in snout-vent length (SVL), 17.9 ± 3.05 cm in tail length (TL), and weighed 47.8 ± 25.4 g. Their most distinctive feature was a vivid green dorsal coloration with well-defined, black maculations that contrasted with a ventral coloration presenting as a pale hue with small dark spots, without defined patterning (Table 1 and Fig. 2). In comparison, *C. subcristatus* hatchlings (*n* = 12) exhibited similar SVL (10.4 ± 1.76 cm; Wilcoxson test, *p* = 0.371) and body mass (35.6 ± 20.3 g; Wilcoxson test *p* = 0.0846), but shorter tail (14.7 ± 1.62 cm; ANOVA, F = 8.752, *p* = 0.00925), resulting in a lower TL/SVL ratio (1.42 ± 0.11 *vs*. 1.65 ± 0.23; ANOVA, F = 8.047, *p* = 0.0119). *C. marthae* neonates’ dorsal surface exhibited a darker, more uniformly black-brown dorsal pattern and light ventral pigmentation with subtle yellow tones (Table 1 and Fig. 2B).

**Table 1:** Morphological and behavioral characteristics of six hatchlings of *Conolophus marthae* and 12 *C. subcristatus*.

Characteristic	*Conolophus marthae*	*Conolophus subcristatus*
SVL (cm)	10.9 ± 1.63	10.4 ± 1.76
Tail length (cm)	17.9 ± 3.05*	14.7 ± 1.62*
Ratio (TL/SVL)	1.65 ± 0.23*	1.42 ± 0.11*
Weight (g)	47.8 ± 25.4	35.6 ± 20.3
Color at birth	Green and black hues	Black and brownish hues
Capture location	Edge and inside the crater	Edge and inside the crater
Dorsal color pattern	Green base with black maculations	Dark brown to black with minimal pattern
Ventral coloration	Pale and unpigmented	Light pigmentation with yellow tones
Behavioral traits	Fast, evasive, difficult to capture	More cautious, less evasive

**Notes:**

Morphological, and behavioral characteristics of six hatchlings of *Conolophus marthae* and 12 *C. subcristatus*, captured during 2022 and 2025 field expedition to Wolf Volcano, Isabela Island, Galápagos.

SVL, snout–vent length.

* Denote interspecific significant (*P* < 0.05) differences. Mean values are given + Standard Deviation.

### Ontogenetic color transition

**Figure 3:**
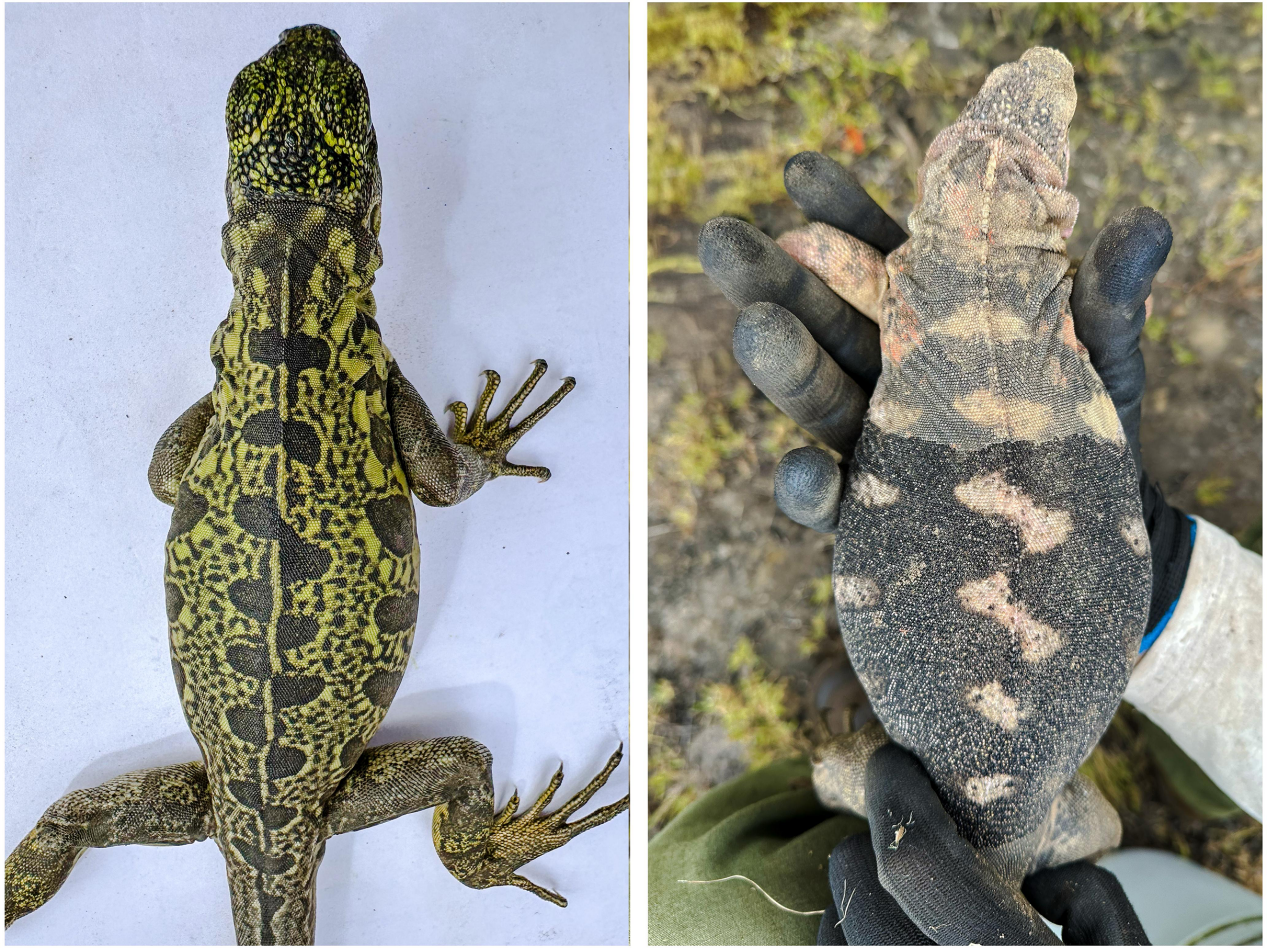
Ontogenetic color transition in *Conolophus marthae*. Ontogenetic color transition in *Conolophus marthae*: comparison between a hatchling (left) and a subadult (right). Green areas in the hatchling become black in the subadult, and black maculations correspond to the pink areas in adults. Pictures taken in the same habitat during the same expedition. (Photo credit: Jorge Carrión-Tacuri).

Comparison of the coloration of a hatchling and a subadult *C. marthae* revealed a directional color shift. The dorsal green tones observed in the hatchling were replaced by matte black in the subadult, while the black maculations in the hatchling corresponded in position to the pink patches in the subadult (Fig. 3) suggesting an ontogenetic shift in chromatophore distribution and pigment expression.

### Behavioral responses

The six *C. marthae* hatchlings all exhibited markedly evasive behavior, characterized by rapid movements, resistance to capture, and frequent attempts to hide—traits consistent with the species’ low detectability in the field. Despite comparable observer effort and similar terrain, only a minority *C. marthae* individuals were successfully captured, as most fled immediately after brief visual contact.

In contrast, *C. subcristatus* hatchlings were encountered more frequently and generally appeared more confident and approachable in the field, facilitating capture. During handling, the 12 *C. subcristatus* hatchlings captured also exhibited a noticeably calmer and less cautious demeanor compared with the highly evasive *C. marthae* hatchlings. The patterns were consistent across individuals and reflect general behavioral tendencies rather than definitive species-level traits, as environmental conditions and observer detections can also influence hatchling responses.

## Discussion

Establishing baseline data from early morphological and behavioral descriptions is essential for monitoring recruitment in wild species. The lack of such information in *C. marthae* highlights the significance of this work, which directly can contribute to conservation efforts for this species. Although our expanded dataset (six *C. marthae* and 12 *C. subcristatus* hatchlings) provides a substantially stronger basis for comparison, these findings should still be considered preliminary. Additional multi-year and multi-cohort sampling will be necessary to evaluate whether the behavioral tendencies, coloration patterns, morphometric differences, and developmental patterns documented here remain consistent across recruitment periods, life-history stages, and environmental contexts.

The green and black dorsal pattern observed in *C. marthae* hatchlings differed markedly from the adult phenotype, suggesting that the species undergoes a defined ontogenetic shift in pigmentation. Ontogenetic color transitions like these are not uncommon among reptiles (Rosenblum, 2005), but they have not been systematically documented in Galápagos land iguanas. Our observations in *C. marthae* suggest that such transitions may occur, although the ecological drivers and habitat associations across life stages remain poorly understood. A possible explanation for this green pigmentation would be an initial ancestral trait that has been maintained throughout its evolutionary history, linked to continental iguanid lineages (López-Delgado et al., 2025). Recent ultrastructural analyses of adult *C. marthae* confirmed the complete absence of iridophores (Scimeca et al., 2024), supporting the hypothesis that these chromatophores may be present early in development and subsequently lost during the transition to the adult pink-and-black phenotype.

Based on these empirical observations, we propose two non-exclusive hypotheses to explain the green dorsal coloration observed in *C. marthae* hatchlings. First, it may represent an ancestral chromatic condition retained in early developmental stages, consistent with the basal phylogenetic position of the *C. marthae* and patterns described in related iguanid lineages. Alternatively, the green hue may confer enhanced crypsis during the short period in warm-wet season of Galapagos when rainfall produces vegetation in both the nesting zone and the summit of Wolf Volcano. However, for most of the year these areas remain open, arid, and dominated by volcanic substrate and sparse xerophytic vegetation, making background matching unlikely. Distinguishing between these scenarios will require integrating colorimetric data, habitat structure, and chromatophore histology across developmental stages.

Another possibility is that hatchlings may occupy the summit and nesting zone only briefly after emergence, later dispersing downslope into intermediate elevations where vegetation persists for most of the year due to the zone’s much higher humidity. In these greener habitats, a green dorsal coloration would offer more effective crypsis advantage than at the summit or nesting zone. This scenario reconciles the observed coloration with a plausible ecological function and highlights the need to investigate early dispersal movements and microhabitat use in *C. marthae*.

Given the basal phylogenetic position of *C. marthae* (López-Delgado et al., 2025) this coloration pattern may reflect the phenotype of ancestral *Conolophus* lineages, as reported in other squamate lineages exhibiting conserved morphological traits (Stayton, 2005), and in amphibians where complex traits have been lost and reappeared (Wiens et al., 2007).

Generally, greenish colors in reptiles are primarily due to an interaction between different types of chromatophores (*i.e*., melanophores, iridophores, xanthophores, and anderythrophores) in skin layers (Bagnara, Fernandez & Fujii, 2007; Teyssier et al., 2015; Rutland et al., 2019), facilitating defense against solar UV radiation aided by the thickness of the stratum corneum and the stratification of the epidermis (Alibardi & Toni, 2006). Although this remains to be confirmed histologically, the complete absence of iridophores in adult *C. marthae* recently reported (Scimeca et al., 2024), suggests the ontogenetic loss of this chromatophore during development and would explain the transition from the green-and-black hatchling phenotype to the pink-and-black coloration of adults. While the functional basis of the adult coloration remains unresolved, one possibility is that dark regions may enhance heat absorption whereas the unpigmented pink areas could facilitate heat dissipation through superficial vascularization, although this hypothesis requires validation through targeted physiological studies.

The greater evasiveness and lower capture success rate of *C. marthae* hatchlings may reflect a stronger selection pressure from predators, particularly from feral cats (Medina et al., 2011). Although the green coloration of *C. marthae* hatchlings may be adaptive in vegetated settings, it could also be detrimental to this species, as it would increase its exposure to predators in the dry, open substrate of Wolf Volcano during much of the year. In such contexts, heightened reactivity might serve as a behavioral compensation for increased detectability. Similar antipredator responses have been documented in other reptiles; for example, hatchlings of the delicate skink (*Lampropholis delicata*) exhibit immediate sprinting and refuge-seeking upon emergence, likely as a survival strategy (Doody & Paull, 2013). Interestingly, the relatively longer tail of *C. marthae* hatchlings compared to that of *C. subcristatus* may provide both mechanical and ecological advantages. A proportionally elongated tail could enhance balance and stability when climbing or moving among shrubs—an ability consistent with field observations of *C. marthae* individuals foraging above ground—while also serving as an effective counterweight during rapid evasive movements on uneven volcanic terrain. Comparative morphometric data across ontogenetic stages indicate that TL/SVL ratios in *C. marthae* are highest at the hatchling stage and gradually converge toward values observed in adults, although remaining consistently higher than in *C. subcristatus*. This pattern suggests that the proportionally elongated tail may be most functionally relevant early in life, potentially supporting climbing ability, balance, and rapid evasive movements during the period of greatest vulnerability. However, further research is needed to assess whether the characteristics observed in *C. marthae* fulfill a specific function in its early stages of life.

These findings contribute directly to ongoing conservation efforts, as the Conservation and Management Plan for *C. marthae* (Rueda et al., 2023) identifies the lack of knowledge on hatchling morphology, distribution, and behavior as a major limitation for recruitment monitoring and management. Furthermore, genomic studies reveal very low genetic diversity and signs of inbreeding, reinforcing the species’ vulnerability (López-Delgado et al., 2025). In turn, our description provides key traits for field identification and lays the groundwork for more accurately assessing age structure in wild populations. Longitudinal studies on growth, survivorship, and pigmentation in *C. marthae*, as well as broader sampling of hatchlings across the population, are necessary to refine conservation strategies. Documenting the early life stage of this critically endangered reptile represents a significant step toward understanding its life history and supporting its recovery in the wild.

## Conclusions

We provide the first morphological and behavioral description of a *Conolophus marthae* hatchlings, filling a critical gap in the species’ early life history knowledge. The distinct green-and-black coloration reliably distinguishes *C. marthae* from sympatric *C. subcristatus* at hatchling. Although some individuals of *C. marthae* exhibited rapid evasive movements during encounters, behavioral responses should be interpreted cautiously given the qualitative nature. Overall, the traits documented here offer practical field characters for identifying hatchlings *in situ* and contribute foundational information for assessing recruitment in wild populations.

A directional ontogenetic shift—from green dorsal pigmentation with black maculations in hatchlings to the pink-and-black phenotype in subadults and adults—suggests a developmental change in chromatophore expression. This may reflect a retained ancestral trait from basal *Conolophus* lineages, although this remains speculative.

Future work should expand hatchling sampling, document intraspecific variation, and examine post-emergence survival. Studies on the adaptive significance of early coloration and its influence on predation risk are also needed.

Given the critically endangered status of *C. marthae*, strengthening our understanding of hatchling trait recruitment dynamics is essential for guiding management actions and reducing the species´risk of extinction.

Understanding this early life stage lays essential groundwork for evaluating age structure, recruitment success, and conservation outcomes. These insights are the basis for ensuring the long-term viability of *C. marthae* in the wild.

## Supplemental Information

10.7717/peerj.20683/supp-1 Supplemental Information 1 Morphometric and behavioral data for *Conolophus marthae* and *Conolophus subcristatus* hatchlings. Snout–vent length, tail length, body mass, coloration, capture location, and behavioral observations for each individual.

## Additional Information and Declarations

### Field Study Permissions

The following information was supplied relating to field study approvals (*i.e*., approving body and any reference numbers):

Galápagos National Park Directorate (GNPD, research permit PC-77-24).

### Data Availability

The following information was supplied regarding data availability:

The raw morphometric data of a hatchling *Conolophus marthae* and sympatric *C. subcristatus* hatchlings recorded during the 2025 field expedition to Wolf Volcano, Galápagos is available in the Supplemental File.
